# An ensemble-based drug–target interaction prediction approach using multiple feature information with data balancing

**DOI:** 10.1186/s13036-022-00296-7

**Published:** 2022-08-08

**Authors:** Heba El-Behery, Abdel-Fattah Attia, Nawal El-Fishawy, Hanaa Torkey

**Affiliations:** 1 Department of Computer Science and Engineering, Faculty of Engineering, Kafrelsheikh University, Kafr_El_Sheikh, Egypt; 2 Computer Science & Engineering Department, Faculty of Electronic Engineering, Menoufia University, Menouf, Egypt

**Keywords:** Drug–target interaction, Data balancing, Support vector machine, Machine learning

## Abstract

**Background:**

Recently, drug repositioning has received considerable attention for its advantage to pharmaceutical industries in drug development. Artificial intelligence techniques have greatly enhanced drug reproduction by discovering therapeutic drug profiles, side effects, and new target proteins. However, as the number of drugs increases, their targets and enormous interactions produce imbalanced data that might not be preferable as an input to a prediction model immediately.

**Methods:**

This paper proposes a novel scheme for predicting drug–target interactions (DTIs) based on drug chemical structures and protein sequences. The drug Morgan fingerprint, drug constitutional descriptors, protein amino acid composition, and protein dipeptide composition were employed to extract the drugs and protein’s characteristics. Then, the proposed approach for extracting negative samples using a support vector machine one-class classifier was developed to tackle the imbalanced data problem feature sets from the drug–target dataset. Negative and positive samplings were constructed and fed into different prediction algorithms to identify DTIs. A 10-fold CV validation test procedure was applied to assess the predictability of the proposed method, in addition to the study of the effectiveness of the chemical and physical features in the evaluation and discovery of the drug–target interactions.

**Results:**

Our experimental model outperformed existing techniques concerning the curve for receiver operating characteristic (AUC), accuracy, precision, recall F-score, mean square error, and MCC. The results obtained by the AdaBoost classifier enhanced prediction accuracy by 2.74%, precision by 1.98%, AUC by 1.14%, F-score by 3.53%, and MCC by 4.54% over existing methods.

## Introduction

Predicting DTIs for prospective drugs plays an essential role in drug discovery. It helps in understanding biological operations and reduces the costs of drug discovery [1, 2]. However, there are many challenges in predicting DTIs. For example, many positive and negative effects of drugs are hard to detect and explain. In the last few years, there have been significant efforts to overcome these challenges and predict DTIs. In addition, because the Human Genome Project has been completed and molecular medicine is being continuously developed, more unknown DTIs have been discovered. However, the number of analytically validated drug–target interactions is still very small, prompting research scientists to devise novel computational approaches to overcome these challenges for potential DTI prediction [3].

An enormous amount of DTI data is produced after the development of high-performing computational technologies. Several popular databases, such as KEGG [4], DrugBank [5], ChEMBL [6], STITCH [7], and TTD [8], that have been created to store confirmed data and to provide relevant recovery information are useful for setting up efficient computational methods for the optimal prediction of DTIs.

Typical DTI computational schemes can be portioned into three categories: ligand-based, simulation docking, and chemogenomic schemes. First, ligand-based schemes utilize target protein similarity to predict interactions between a drug’s chemical structures and protein sequences [9].

Second, docking-based schemes use dynamic imitations of a target protein to discover novel, unknown interactions. Such schemes are a prospective technology that enforces the 3D structure of proteins to address the prediction stage [10].

Chemogenomic schemes establish a prediction model depending on graph theory [11, 12], network methods [13, 14], and techniques based on machine learning [15, 16]. Among the chemogenomic approaches, machine-learning approaches are regarded as the most dependable for predictive outcomes. Machine-learning approaches can be categorized into features or similarity method.

Similarity techniques have been developed to calculate the similarity among drug compounds and target proteins [17, 18]. Similarity-based techniques contain matrix factorization [13], kernel-based approaches, and graph-based approaches [11].

Feature methods represent target–drug pairs with a vector with a carrier of prescriptions. Different properties of target–drug pairs have been coded as related features. In feature techniques, the DTIs are predicted by detecting the most distinct features. Hence, the inputs to these techniques are different vectors resulting from a combination of the properties of drugs and targets. These vectors have been computed by specifying a coding characteristic or bioinformatics software package that can perforce calculate its chemical and biological characteristics. Because these vectors usually have many dimensions, some methods use dimensionality reduction approaches to decrease the number of features, thus improving the performance model and prediction efficiency.

In drug–target interaction prediction, many types of features were used for both drugs and targets, such as in [19], where the authors used drug feature vectors of constitutional, topological, and geometrical descriptors. The protein features used are amino acid, pseudo amino acid, and composition, transition, and distribution (CTD) descriptors. In addition, [20] used Morgan molecular fingerprints for the drug feature vector, and the protein feature was 20 amino acids. There are many medical libraries used to find these features, such as the RDKit library [21], RCPI library [22], and PyBioMed library [23].

Several ML techniques such as XGBoost [24], deep learning [16], support vector machine (SVM) [25], and nearest neighbor are used for discovering possible DTI features more effectively.

We are developing a framework for DTI prediction that uses the most popular drug-molecular fingerprinting, Morgan fingerprints [26], also known as ECFP4 extended conduction fingerprints. Morgan fingerprints have been generated as binary. Morgan fingerprints are often used in the predictive modeling of bioactivity to allow meaningful chemical diffusion to be decoded into the chemical space.

The secondary characteristic of drugs is its constitutional descriptors, which are the easiest molecular descriptors that can be calculated from the molecular structure. Constitutional recipes include all those representing a molecular structure, which regards only the chemical structure and does not encode information regarding topology and general geometry.

We apply the most common property for proteins, which consists of long chains of α-amino (alpha-amino) acids [27]. The AAC knows the number of amino acids of each type normalized with the overall number of residues.

The secondary feature of proteins is the dipeptide composition [28, 29], which is useful over simple AAC, which provides a composition of a pair of residues present in the peptide. Dipeptide composition constitutes a better feature than AAC as it encases the information of both amino acid fraction and the local sort of amino acids.

In this paper, we presented a DTI prediction model dependent on the drug chemical structures and protein sequencing of trait extraction using a medical library. We developed an approach to predict negative samples using an SVM one-class classifier to overcome the imbalance problem between negative and positive samplings and then built four feature sets from the negative and positive sampling drug–target datasets. Finally, these feature sets were imputed into the prediction algorithm to determine the DTI.

The major contributions in this paper could be summarized as follows:

i. An approach for predicting negative samples using an SVM one-class classifier for handling imbalance problems between negative and positive samplings that had not been effectively addressed in existing approaches was developed.
ii. Four feature sets from the four types of drug–target features and the negative and positive samples were constructed. Then, these feature sets were applied to various types of machine-learning algorithms to predict DTIs.
iii. The proposed approach was compared to existing models, indicating the superiority of the proposed model by achieving the best performance scores across the DrugBank dataset. The results of the proposed model outperformed recent research in the field of DTI. The proposed model obtained an average accuracy 2.74% higher than that of recent studies and AUC, F-score, and MCC of 1.14, 3.53, and 4.54%, respectively.
iv. Propose the feature analysis using feature importance and data set balancing.

This paper is structured as follows. In Section 2, existing related methods of DTIs are presented. Our proposed framework, together with a detailed description of the used techniques and datasets, is presented in Section 3. In Section 4, the results and discussion are provided. The feature analysis, data balancing and comparison with the latest methods: are presented in Section 5,6. Finally, the conclusion is described in Section 7.

## Related work

In recent years, several approaches using machine-learning algorithms have been elaborated for DTI prediction initiatives. In general, first, a library was used to extract the drug and target features from the input data. Then, positive and negative samples were identified and then inputted into prediction methods. Finally, the model was evaluated using evaluation matrices.

Table 1 shows that DTI-SNFRA  [30] works in two phases: first, it uses an SNN, followed by a search space-partitioning group, and then, it calculates the degree of fuzzy-raw approximation and selects the appropriate degree threshold for excitation samples’ undercounting from all possible drug–target interaction pairs obtained in the first stage. In [31] and [16] the deep learning structures models discovered local survival patterns the target successfully enriches protein advantages of the raw protein sequence, leading to greater predictive results than related approaches. In [32], the authors presented a multi kernel-based learner along with decreased features and extracted prediction scores to indicate the results, while The authors in [33] developed a FastUS algorithm was used to overcome the class imbalance constraint. The authors in [20] presented a method for DTI prediction using LOOP and Matrix (PSSM). In particular, LOOP is used for extracting feature vectors from PSSM. By contrast, the authors in [34] used the features tested with the (E-state) fingerprints of the drug smiles and (APAAC) of the protein sequences. In [35], the authors developed a new heterogeneous multi molecule information network created by a combination of n-known connections between proteins and drugs.

**Table 1:** Summary and comparison of DTI prediction methods for identification interactions relative to our presented framework

Paper	Drug feature and protein feature	Method for negative samples	Description	Method
DTI-SNNFRA [30] (2021)	**Drug:** constitutional, topological, and geometrical descriptors. Protein: amino acid, pseudoamino acid, and CTD	First is the similarity between the drugs and the proteins. Then, the shared nearest neighbors and k-medoids clustering	First, the similarity between the drugs and the proteins. Then, the shared nearest neighbors and k-medoids clustered using the RUSBoost classifier for the prediction stage.	1. Shared nearest neighbors 2. RUSBoost Classifier
DeepCon [31] (2019)	**Drug:** Morgan fingerprint **Protein**: CNN on raw protein sequence, CTD	Dependent on the similarity between the drugs and the proteins; then compute the distance between the drug and protein.	First compute the distance depending on the similarity of drug and target features for predict the negative samples to achieved the class balance, second apply to DBN for prediction stage.	1. The similarity of drug and target features 2. Deep belief network (DBN)
Idti-MLKdr [32] (2021)	**Drug:** Morgan fingerprint **Drug:** AAC, DC, TC	evaluate the molecular similarity of drug and target features based on the Tanimoto coefficient (TC). Then, the Cluster-Based Molecular Similarity algorithm calculates and selects the top-ranked drugs and targets.	The Tanimoto coefficient (TC) depends on the similarity between the drugs and between the proteins. Then, use Cluster algorithm and finally using Multikernel learning (MKL).	1. Cluster algorithm 2. Multikernel learning (MKL)
PreDTIs [33] (2021)	**Drug:** drug-molecular substructure pattern fingerprint **Protein**: Psepssm	Using the SVM classifier. Then, the Euclidean distance is calculated from the predicted and the value of the real features	Use the SVM classifier. Then, calculate the Euclidean distance between the real and predicted values, using the LightGBM for prediction.	1. Euclidean distance 2. LightGBM Classifier
[20] (2020)	**Drug:** molecular substructure fingerprints **Protein**: Apply the PSSM, and then, apply the LOOP method to extract protein feature	Randomly select the number of negative samples, which is the same as the number of positive samples.	Randomly select the negative samples, equal to the positive samples. Apply the rotation forest for prediction.	1. Rotation forest
[35] (2020)	**Drug:** Morgan fingerprint. **Protein**: 20 amino acids	The negative sample sets consist of the same number of randomly selected pairs of unrelated drugs and proteins.	Randomly select the negative samples. Apply Random Forest for prediction.	1. Random Forest classifier
[16] (2017)	**Drug:** molecular descriptors and molecular fingerprints (MFs). **Protein**: AAC, DC, and TC	The negative dataset can be randomly selected from the DTS.	Random select the negative samples. Apply the deep belief network for prediction	1. Deep belief network (DBN)
[34] (2020)	**Drug:** (E-state) fingerprints **Protein**: (APAAC)	The Euclidean distance from all unlabeled samples to the positive center is calculated and sorted. The farther the distance is, the more likely the sample is to be negative.	The Euclidean distance from all unlabeled samples to the positive center. Apply support vector machines (SVM) for prediction.	1. Euclidean distance 2. Support vector machines (SVM)

## Materials and methods

### Proposed model overview

**Fig. 1:**
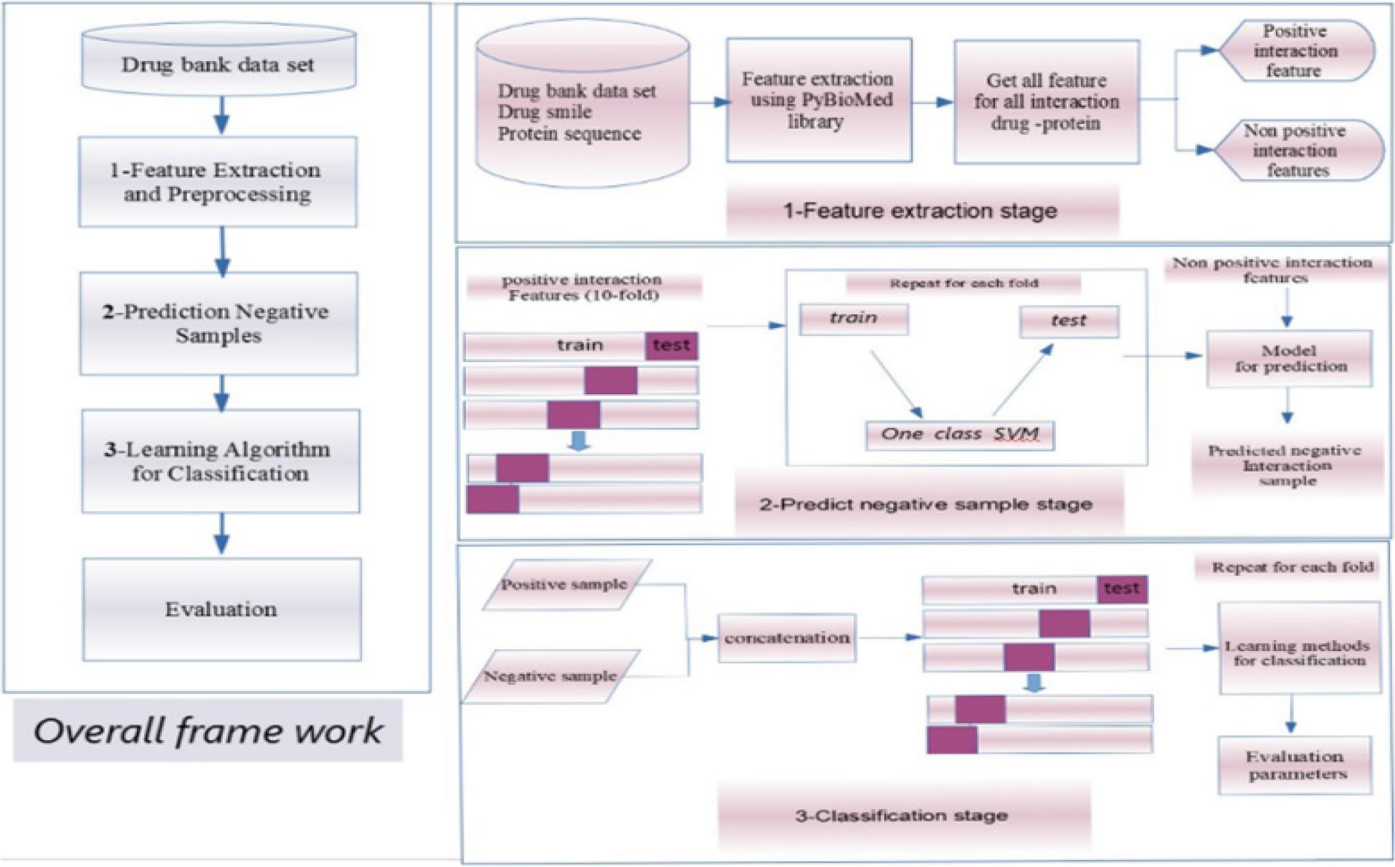
The proposed framework model: A) is the overall prediction framework, 1) is the feature extraction and preprocessing stage for the DTI dataset, 2) is the prediction of negative samples stage, and 3) is the application of the prediction algorithms stage

The schematic diagram of the presented framework method is shown in Fig. 1. Initially, the drug structures (SMILE format) and protein sequences (FASTA format) were aggregated from DrugBank databases using access identifiers. Various feature extraction techniques were applied to drug and protein sequences to generate different features. Features using a single row SVM and known interaction to predict negative samples. Ultimately, the framework was trained using prediction algorithms to classify the four feature sets and evaluate these algorithms.

#### Feature extraction

The drug input was represented as a Simplified Molecular Input Line Entry System, which described the structure of chemical species using short ASCII strings. Drug SMILE, which included full chemical structure information, was aggregated from the DrugBank databases by its specific drug ID.

This article used the PyBioMed Software Toolkit [23], which is a responsive feature-rich python application for manipulating chemical structures in different file formats, permitting them to be analyzed, converted, and stored. PyBioMed [23] can produce 18 kinds of molecular fingerprints.

In this study, the first drug feature was Morgan fingerprints because it enhances the efficiency of research and analysis of drugs. For representing drug properties, the SMILE format was transformed to Morgan, where the molecular fingerprint pattern was a digital sequence of 1024 digits. The 1024-dimensional feature vector was derived from each pharmacological chemical structure.

The second drug features were constitutional descriptors, which are the simplest and most used descriptors that reflect the chemical structure of a compound without information regarding its molecular geometry or atom connection. The 30-dimensional feature vector was obtained from the chemical composition of a compound.

For the proteins, features that were extracted from the protein sequences from the FASTA format were collated from the DrugBank database using the PyBioMed Software Toolkit [23] to derive the target features from the protein sequences. These features incorporate amino acid composition (AAC) and dipeptide composition (DC). AAC involves 20 elements, each of which is one of the 20 amino acids in the protein sequence. Dipeptide composition (DC) considers the fraction of every two AAC residues in the protein sequence. The DP captures protein sequence order information in pairs, which is the main feature. DP provides 400 features.

#### Negative sample prediction

In the dataset section, the number of unknown interactions was 58,629,134. Then, we constructed the unknown interaction feature set. This is a major problem in storing and processing, so we tried to present a new proposal schema in these interactions to overcome data balancing.

One-class SVM is an unsupervised algorithm for learning the decision function of novel discovery: predicting new data as identical or distinct to the training package. The one-class SVM algorithm is constructed by assessing a probability distribution function that determines the distance of most data on hyperplane. A decision rule separates these observations by the most significant potential margin [36]. The computational complexity of the learning phase is intense because one-class SVM training involves a quadruple programming problem. Once the decision function is defined, it can predict the stratified mark of new test data.

**Fig. 2:**
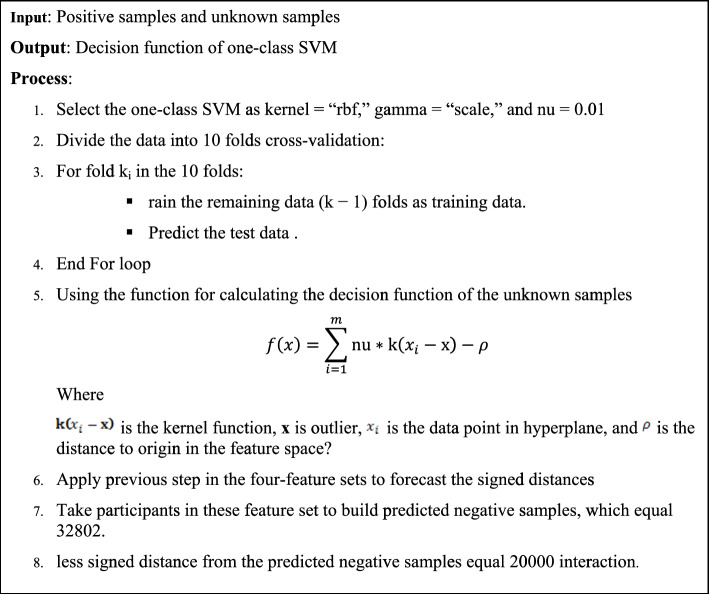
The pseudocode to predict negative samples using a one-class SVM classifier

Figure 2 provides the procedure used to predict the negative samples using a one-class SVM classifier.

We developed an approach for predicting negative samples using a one-class SVM classifier. This algorithm works too.

1. Determine all unknown interactions (equal to 58,629,134 interactions).
2. Use the one-class support vector machine-learning algorithm for classifying the positive samples into a hyperplane, which is executed on 10-fold cross-validation. The empirical feature set is split into training and testing feature datasets. In addition, it uses to predict the signed distance for unknown interaction from the positive hyperplane.
3. Apply the previous step in the four feature sets to forecast the signed distances, which are the distances of all samples to the separating hyperplane learned by the model.
4. Take the participants in these feature sets to build predicted negative samples equal to 32,802. Then, we sort these samples to get the less signed distance for predicted negative samples.

Finally, we constructed the feature sets from the table using the positive and negative interactions (39,866 interactions). The pseudocode for this algorithm is shown in Fig. 2.

#### Prediction approaches

Our previous work [15] demonstrated that the ensemble learning-based algorithms for DTI predictions are most accurate for predicting drug–target interactions. These ensemble-learning algorithms were employed in this paper and were compared with other machine-learning algorithms.

Five different prediction algorithms were used: RF, AdaBoost, XGBoost, Light Boost, and SVM. Drug–target feature sets were roughly separated into ten subgroups by a 10-fold CV validation test. One of the ten groups was selected as a test group, the remaining nine were considered a training group, and this operation (cross-validation) was repeated 10 times. After calculating the average of the 10 verification results, the results were created from the drug–target datasets using deferent types of prediction algorithms.

- Support vector machine (SVM)

SVM is an honorable machine-learning method that can be used for concurrent prediction and regression problems. The prediction is performed by identifying the plane that characterizes the most for each category of data. In this method, SVM parameters are {reg_p = 1.0, kn = ‘rbf,’ gama = ‘scale’}.

The parameters are as follows:

- reg_p: It is the regularization parameter.
- kn: It specifies the kernel type to be used in the algorithm. The default value is “RBF.”
- gama: It is the kernel factor

b) Random Forest (RF)

RF is an ensemble-learning technique for prediction. RF works well for a wide scale of data elements from a single decision tree. In addition, a precision RF algorithm can be maintained even with a large percentage of data missing. The parameters of this technique are {max feature = 0.3, min samples split = 16, num of estimators = 115}.

The parameters are as follows:

- max feature is the max number of random most fore features considers splitting a node.
- min samples split is the minimum number of leaves required to split an internal node.
- num of estimators are several trees that the algorithm builds before taking the maximum voting or taking the averages of predictions.

- AdaBoost

Adaptive Boosting is the weights redistributed to each condition, with the highest weights assigned to incorrectly ranked cases. Adaptive Boosting is a good ensemble technique widely used for concurrent prediction and regression problems. The parameters used in this method are {splitter = ‘best,’ max depth = 6, min samples split = 2, algorithm = “SAMME,” number of estimators = 90}.

The parameters are as follows:

- min samples split is the minimum number of leaves required to split an internal node.
- num of estimators are several trees that the algorithm builds before taking the maximum voting or taking the averages of predictions.

Algorithm: use the SAMME discrete boosting algorithm.

Splitter: strategy used to choose the split at each node.

Max depth: the max depth of the tree.

b) XGBoost

XGBoost optimizes the ensemble model depending on gradient tree boosting, which is widely used in prediction tasks. The parameters used in this method were {max_depth equal to 5, learning_rate equal to 0.2612, n_estimators equal to int (75.5942), reg_alpha equal to 0.9925, thread equal to − 1, objective equal to ‘binary: logistic’}.

iii) Light Boost

Light Boost is a fast, high-performance unitary technique that uses distribution technique like the decision tree algorithm. The parameters used in this method were learning rate = [0.001, 0.01, 0.1, 0.2, 0.3], momentum number = [0.0, 0.2, 0.4, 0.6, 0.8, 0.9], optimizer method = SGD, objective = binary, and boosting = gradient boosting.

## Evaluation parameters

The different measures used for drug–target interaction prediction for evaluating and comparing different techniques are [15] as follows:

$$Accuracy=\frac{TP+ TN}{\left( TP+ TN+ FP+ FN\right)},$$

$$Precision=\frac{TP}{\left( TP+ FP\right)},$$

$$Recall=\frac{TP}{\left( TP+ FN\right)},$$

$$F1\ Score=\frac{2\ast \left( Recall\ast Precision\right)}{\left( Recall+ Precision\right)},$$

$$mcc=\frac{TP\ast TN- FP\ast FN}{\sqrt{\left( TP+ FN\right)\ast \left( TN+ FP\right)\ast \left( TP+ FP\right)\ast \left( TN+ FN\right)}},$$

where TP is true positive, TN is true negative, FP is false positive, and FN is false negative.

The area under the curve:

The receiver operating characteristic (ROC) curve displays the performance of the forecaster with different threshold values.

### Mean squared error (MSE)

MSE calculates the average of the squares of the errors.

$$MSE=\frac{1}{n}\sum_{i=1}^n{\left({\mathrm{Y}}_{\mathrm{i}}-\hat{Y_i}\right)}^2.$$

## Results and discussion

In this section, we underline the effective results of our DTI prediction model that implements the four feature sets. Each technique is applied in python language by sci-kit-learn, ensemble package, Kares library, TensorFlow library, and XGBoost package (version 3.8). The algorithms were sped up using Windows 10 with a 3.10 GHz Intel core i9 processor and 64.0 GB RAM.

### Dataset

The empirical drugs and targeted datasets were aggregated from the DrugBank [5] database. The DrugBank database includes SMILE chemical structures and FASTA sequences with certified, experiential, nutraceutical, biotech, and withdrawn version (Group) drug and protein packages. Our study’s approved version of drugs, targets, and interactions of experimental datasets is on the recent release of DrugBank Online (version 5.1.8, released 2021-01-03). Our datasets consist of 11,150 drugs and 5260 protein targets with 58,649,000 potential interactions, with just 19,866 interactions noted as positive interactions as shown in Table 2. Thus, the number of positive interactions is much lower than that of the potentially negative interactions. The number of unknown interactions is equal to 58,629,134, causing an imbalance in the datasets. For this reason, we presented a method for predicting the negative samples to dominate the imbalance between positive and negative interactive datasets. The DrugBank dataset statistics are presented in the DrugBank database.

**Table 2:** DrugBank dataset statistics

Drug	Protein	Positive interaction
11150	5260	19866

We applied these datasets to feature generation processes and extracted the features. These features combined the four feature sets of the interaction between the drug and protein. The different combinations of these feature sets are shown in Table 3.

**Table 3:** Four feature sets of the drug–target interaction

Feature set	Drug feature	Protein feature	Number of features
Feature set [1]	Morgan fingerprint	Amino acid composition	1044
Feature set [2]	Morgan fingerprint	Dipeptide composition	1424
Feature set [3]	constitution	Amino acid composition	50
Feature set [4]	constitution	Dipeptide composition	430
All feature set	Morgan fingerprint + constitution	Amino acid composition + Dipeptide composition	1474

Now, we have five feature sets with a different number of features.

### The results for negative sample prediction

SVM one-class learning requires the selection of the *kernel* and the stable coefficient to define the boundary. An RBF kernel is usually chosen even though there is no exact formula or algorithm for determining the bandwidth factor. The second important parameter in SVM one-class learning is a *nu* parameter, known as the one-order SVM margin, which corresponds to the possibility of finding a new, but regular, observable out-of-bounds nu that is equal to 0.01.

First, in the one-class SVM, training with positive samples to construct the hyperplane in all positive samples (positive hyperplane) occurs. Then, using the decision function in this method, determine the distances between the unknown interactions and the positive hyperplane. Next, apply this function in four feature sets. Second, determine the highest negative value of the distances, which indicates the highest outliers from the positive hyperplane. The evaluation results are shown in Table 4.

**Table 4:** Evaluation results of negative sample prediction using one-class SVM

Method	Precision	Recall	F-score	Accuracy
One-class SVM	1	0.989	0.995	0.989

### The prediction algorithm results

The results in Table 5 record the accuracy, mean square error, MCC, and F-score obtained by different techniques. Using feature set [1], the highest accuracy score value of 0.9999 is achieved by AdaBoost ensemble learning, and Light Boost obtained the second best value of 0.9998.

**Table 5:** Evaluation results of feature sets of the drug–target interaction using machine and ensemble algorithms according to precision, recall, F-score, and accuracy

Feature set	Prediction algorithms	Precision	Recall	F-score	Accuracy
Feature set [1]	SVM	0.995	0.995	0.995	0.996
	RF	0.9996	0.9996	0.9996	0.9997
	AB	**0.9998**	**0.9998**	**0.9998**	**0.9999**
	XG	0.9994	0.9995	0.9995	0.9996
	Light	0.9997	0.9997	0.9997	0.9998
Feature set [2]	SVM	0.9992	0.9992	0.9992	0.9991
	RF	0.9996	0.9996	0.9996	0.9996
	AB	**0.9998**	**0.9998**	**0.9998**	**0.9998**
	XG	0.9995	0.9995	0.9995	0.9996
	Light	0.9996	0.9996	0.9996	0.9997
Feature set [3]	SVM	0.992	0.992	0.992	0.992
	RF	**0.9993**	**0.9993**	**0.9993**	**0.9992**
	AB	**0.9993**	**0.9993**	**0.9993**	**0.999**
	XG	0.999	0.999	0.999	0.9988
	Light	0.9989	0.9989	0.9989	0.9987
Feature set [4]	SVM	0.951	0.948	0.948	0.942
	RF	0.999	0.999	0.999	**0.9989**
	AB	**0.9992**	**0.9992**	**0.9992**	**0.9989**
	XG	0.999	0.999	0.999	0.9987
	Light	0.9988	0.9988	0.9988	0.998
All Feature set	SVM	0.993	0.993	0.993	0.994
	RF	0.9992	0.9992	0.9992	**0.9993**
	**AB**	**0.9993**	**0.9993**	**0.9993**	**0.9993**
	XG	0.998	0.998	0.998	0.998
	Light	0.9991	0.9991	0.9991	0.999

For feature set [2], the highest precision score value, best recall value, highest F-score value, and highest accuracy score value of 0.9998 were achieved by AdaBoost ensemble learning and Random Forest. Light Boost obtained the second highest value of 0.9996.

For feature set [3], the best precision score value, best recall value, best F-score value, and highest accuracy score value of 0.9993 were obtained by AdaBoost ensemble learning and Random Forest. XGBoost obtained the second highest value of 0.999.

For feature set [4], the best precision score value, best recall value, best F-score value, and highest accuracy score value of 0.999 were obtained by AdaBoost ensemble learning and Random Forest. SVM obtained the worst value for prediction.

For all feature sets, the best precision score value, best recall value, best F-score value, and highest accuracy score value of 0.9993 are obtained by AdaBoost ensemble learning and Random Forest, and SVM obtained the worst value for prediction.

From the previous results, it was found that feature sets 1 and 2 gave better results than the others because they contained a representation of drugs using Morgan’s fingerprint. This gives support that Morgan’s fingerprint is a better representation of drugs than the other features used. When all features were used, we found a decrease in the results, which means that some features do not give a good description of drugs and proteins. In drug features found constitutional descriptors achieve the worst results in DTIs prediction.

The results are in Table 6. record area under the curve (AUC), mean square error, and MCC achieved by different techniques. Using feature set [1], the highest AUC value of 0.9998 was obtained by AdaBoost ensemble learning, and Light Boost obtained the second best value of 0.9997. The best MCC value of 0.9996 was obtained by AdaBoost and Light Boost ensemble learning.

**Table 6:** Record area under the curve (AUC), mean square error, and MCC are achieved by different techniques

Feature set	Prediction algorithms	AUC	Mean square error	MCC
Feature set [1]	SVM	0.9954	0.0047	0.99
	RF	0.9996	0.00038	0.9993
	AB	**0.9998**	**0.00023**	**0.9996**
	XG	0.9995	0.0005	0.9991
	Light	0.9997	0.0003	**0.9996**
Feature set [2]	SVM	0.981	0.0008	0.998
	RF	0.9996	0.00035	0.9993
	AB	**0.9998**	**0.00015**	**0.9997**
	XG	0.9995	0.0004	0.9994
	Light	0.9996	0.0004	0.9991
Feature set [3]	SVM	0.976	0.0082	0.984
	RF	**0.9993**	**0.0007**	**0.9986**
	AB	**0.9993**	**0.0007**	**0.9986**
	XG	0.999	0.0009	0.9982
	Light	0.9989	0.001	0.9979
Feature set [4]	SVM	0.949	0.051	0.8997
	RF	0.999	0.0009	**0.998**
	AB	**0.9992**	**0.0008**	**0.998**
	XG	0.999	0.0009	**0.998**
	Light	0.9988	0.001	0.997
All feature sets	SVM	0.993	0.007	0.986
	RF	0.9992	0.0008	0.999
	**AB**	**0.9993**	**0.00067**	**0.999**
	XG	0.998	0.0018	0.996
	Light	0.9991	0.00085	0.998

For feature set [2], the best AUC value and best MCC value of 0.9998 and 0.9997, respectively, were obtained by AdaBoost ensemble learning. Random Forest and Light Boost obtained the second highest value of 0.9996.

For feature set [3], the best AUC value and best MCC of 0.9993 and 0.9986, respectively, were obtained by AdaBoost ensemble learning and Random Forest. XGBoost obtained the second highest value of 0.999.

For feature set [4], the best AUC value and best MCC value of 0.999 and 0.998, respectively, were obtained by AdaBoost ensemble learning, Random Forest, and XGBoost. AdaBoost ensemble learning also obtained the least mean square error for prediction.

For the all feature set, the best AUC value and best MCC value of 0.9993 and 0.999, respectively, were obtained by AdaBoost ensemble learning. In addition, AdaBoost ensemble learning provided the least mean square error for prediction.

The AUC is computed depending on every model’s AUC curve for describing the quality of work, which offers the most accurate visual explanation for predicting DTIs.

**Fig. 3:**
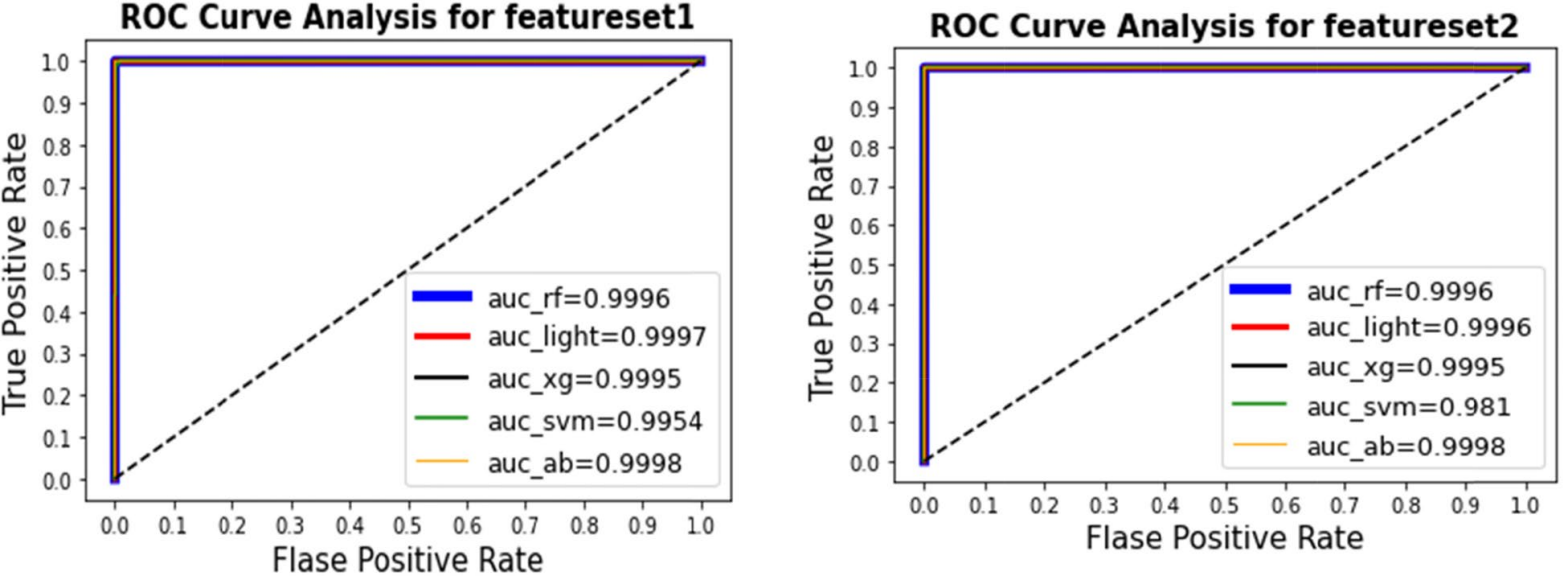
The results for the ROC curve and the value of AUC for the learning techniques show that the AdaBoost method predicts the max score in the AUC = 0.9998 for feature set [1] and set [2]

**Fig. 4:**
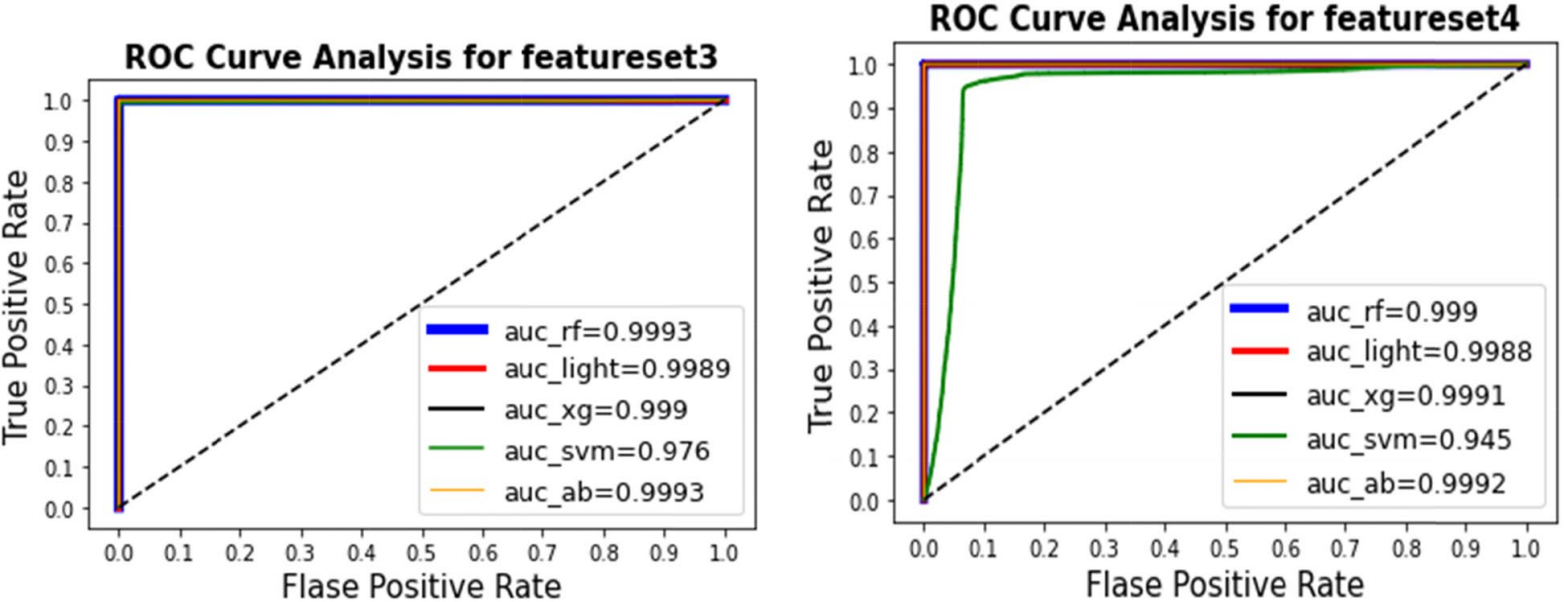
The results of the ROC curve and the AUC value for the AdaBoost and Random Forest learning methods, which predicted the max AUC as 0.9993 for feature set [3]. In feature set [4], the AdaBoost method predicted the max score in the AUC = 0.9992

**Fig. 5:**
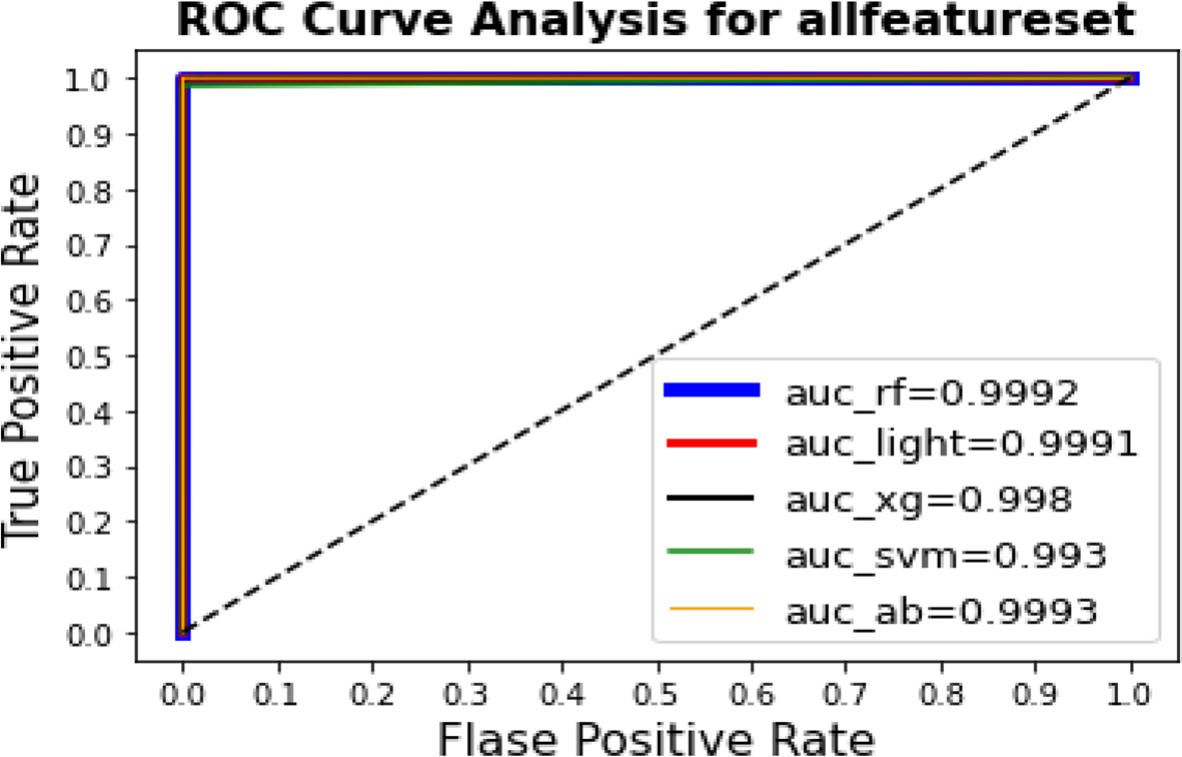
The results of the ROC curve and the value of the AUC for the learning techniques. The AdaBoost method predicted the max score in the AUC = 0.9993 for all feature sets

Figure 3 shows the ROC curve and value of AUC for the learning techniques. Using feature set (1), the best AUC value of 0.9998 was obtained by AdaBoost ensemble learning. For feature set (2), the best AUC value and best MCC value of 0.9998 were obtained by AdaBoost ensemble learning. Figure 4 shows the ROC curve and value of AUC for the learning techniques. For feature set (3), the best AUC value of 0.9993 was obtained by AdaBoost ensemble learning and Random Forest. For feature set (4), the best AUC value of 0.999 was obtained by AdaBoost ensemble learning.  Figure 5 shows the results of the ROC curve and the value of the AUC for the learning techniques. The AdaBoost method predicted the max score in the AUC = 0.9993 for all feature sets

The best results were obtained with the classifier because one of the defects of the classifier is that it is sensitive to outlier samples. This indicates that a very large proportion of the outlier samples had been removed to give the best using our methods in predicting negative samples using a one-class SVM classifier.

### Feature analysis

#### Feature importance

In the study, we applied machine learning to discover the important features from different types of features that are used. The genetic algorithm [37] and XGBoost are the methods chosen because they obtain the highest performance compared to other methods.

**Fig. 6:**
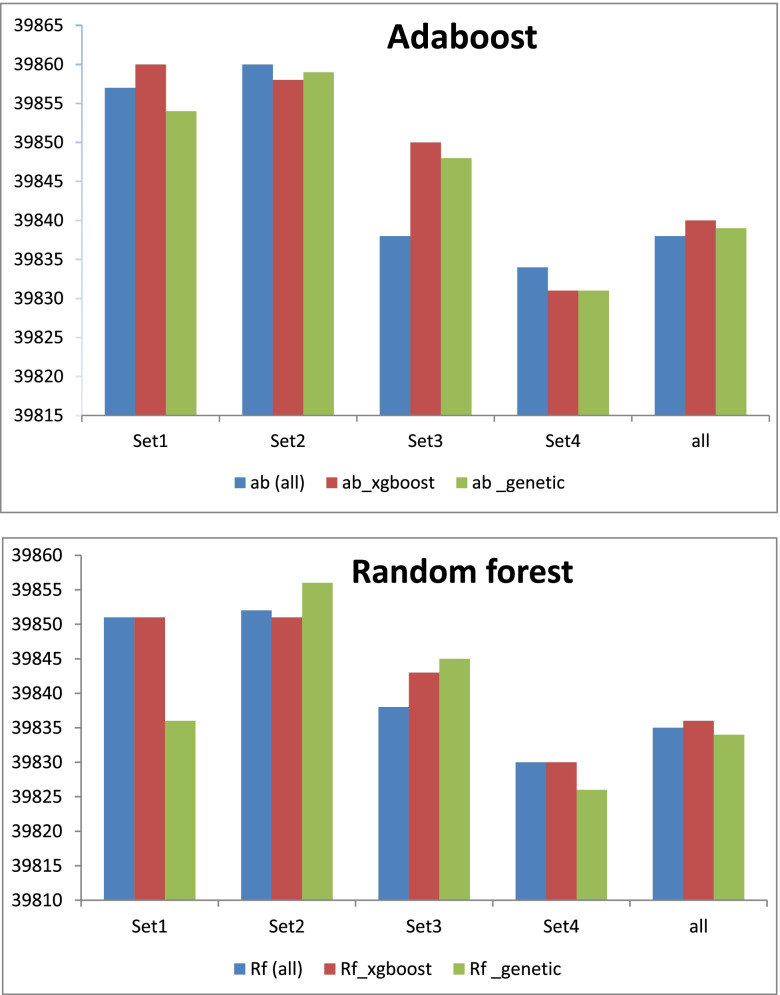
The results when applying the feature important stage before the classifier showed that the XGBoost method obtained the highest score for feature set [2] in the Random Forest classifier whereas the genetic method obtained the highest score in feature set [1] in the AdaBoost classifier

Figure 6 shows the number of correctly classified samples in different learning techniques. Using Random Forest, the best number of correctly classified samples is obtained by the genetic method in feature set [2] and feature set [3]. For AdaBoost, the best number of correctly classified samples is obtained by XGBoost ensemble learning in feature set [1], feature set [3], and all feature set.

#### Undersampling and oversampling methods

In our study, we applied under sampling and oversampling methods for comparison with the proposed model that used the random under sampling technique for under sampling methods [38] and the SMOTE technique for the oversampling method [38].

Our approach exceeded all other under sampling and oversampling methods because we relied on predictions of negative samples by assessing a probability distribution function in one-class SVM.

**Fig. 7:**
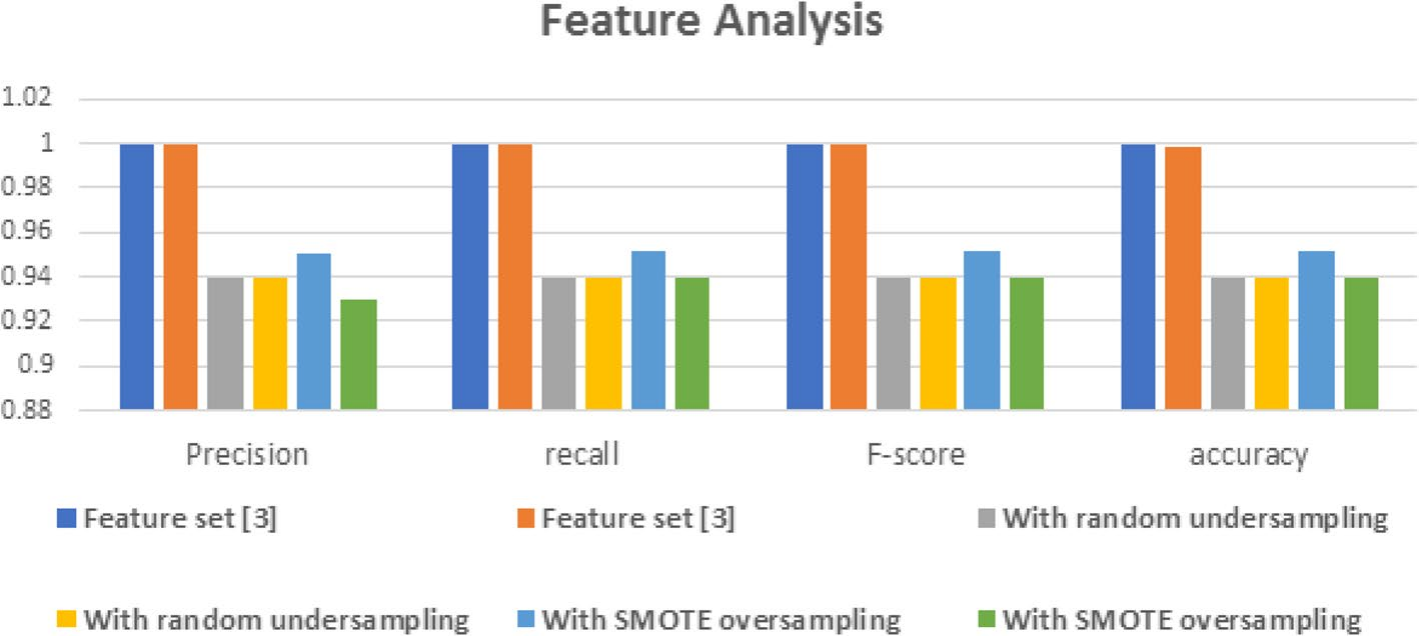
The results when applying the feature analysis stage using the random under sampling and SMOTE oversampling method in feature set [3] and using the Random Forest and AdaBoost obtained the highest performance in all feature analyses

Figure 7 shows that our approach exceeded the best performance in different learning techniques. Using Random Forest and AdaBoost, in feature set [3]. Finally, we calculated the bias of the roads, and the average value was 0.249.

## Comparison with the latest methods

**Fig. 8:**
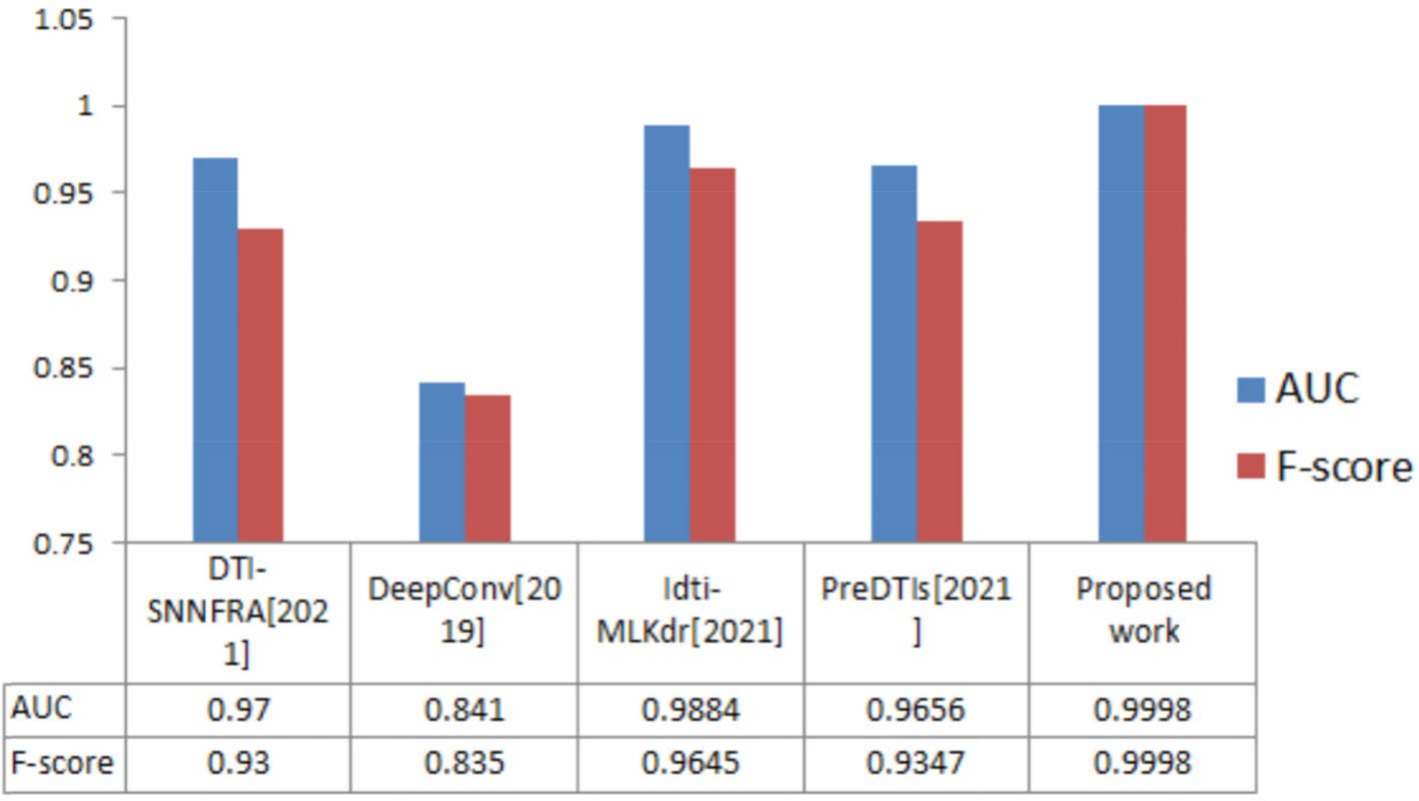
The comparison between related works and the proposed work (feature set [2])

Our framework was compared with four methods [30–33], and the results are shown in Fig. 8. Our approach outperformed all others by achieving the highest performance across the DrugBank, especially in feature set [2]. As shown in Fig. 8, our framework (highest average accuracy = 0.9997) has a 2.74% higher average accuracy than the model in [32], 10.98% higher average precision than the model in [31], and 1.14, 3.53, and 4.54% higher average in AUC, F-score, and MCC, respectively, than the model in [32].

Our model obtained the best results [31, 32] because we operated a one-class SVM to determine the negative and positive samples, which gave better results than using the clustering algorithm in [32]. In addition, we used it at the prediction stage, and we have proven in previous research that ensemble learning obtained the best performance.

## Conclusion

Our study presented a new computational framework for predicting DTIs using the DrugBank dataset. There are two critical challenges in this field: 1) the vast amount of drug and target interactions that create a wide area of research and 2) the imbalanced dataset for DTIs because there are very few DTIs that have been detected so far. For this reason, the size of the negative samples is considerably larger than that of the positive sample. The contributions of this paper are the determination of negative samples for effective prediction and the study of the effectiveness of chemical and physical features in the evaluation and discovery of the drug–target interactions.

We have discovered that the process of predicting negative samples using one-class SVM may be the best in selecting negative samples found in all samples that have not yet been detected. In addition, we have discovered that features, such as Morgan fingerprint and dipeptide composition, in feature set 2 are the best in a characterization process. The performance of the presented method in the prediction stage is largely accurate in DTI prediction, especially when comparing various predictions. The presented method showed strength and stability in DTI prediction.

We have faced the problem of time and processing power while detecting drug–target interactions. We have overcome the lack of processing power using a computer device with special specifications to complete the work, but we still have the problem of time. We suggest using reconstruction methods whole reconfiguring data to improve the performance of lower quality data.

## Data Availability

All data generated or analyzed during this study are included in this published article.
